# Engagement marketing for social good: Application to the *All of Us* Research Program

**DOI:** 10.3389/fgene.2022.889195

**Published:** 2022-08-08

**Authors:** Megan A. Lewis, Jennifer D. Uhrig, Elizabeth T. Adams, Jill A. Brown, Amy Sanders, the RTI International All of Us Innovator Team

**Affiliations:** Center for Communication Science, Translational Health Sciences Division, RTI International, Research Triangle Park, NC, United States

**Keywords:** engagement marketing, social good, *All of Us* Research Program, human-centered design, co-creation

## Abstract

Engagement marketing, when applied to increasing the social good, involves making a deliberate effort to engage communities with an organization’s brand that might not have otherwise happened organically. Organizations that typically focus on increasing the social good include non-profits, community organizations, public health departments, and federal, state, and local agencies. Engagement marketing builds relationships, gives a voice to, and fosters collaboration with community members to transform their insights into impactful experiences that motivate and empower them to act to increase the social good. These actions may include making an informed decision, changing a health or prosocial behavior, or joining an effort that promotes or increases social good. In this paper, we translate the commercial engagement marketing approach, typically used, and studied widely to increase profits, to one that uses engagement marketing to increase prosocial outcomes. We propose a new definition of engagement marketing applied to the social good, a multi-level conceptual framework that integrates individual, social, community and macro-level processes and outcomes, and illustrates an example applying this translated model to co-create digital engagement experiences using a human centered design approach for the *All of Us* Research Program. This model can also guide research and practice related to DNA-based population screening.

## Introduction

Medicine and public health are at an inflection point in which advances in the collection, management and analysis of big data have the potential to lead to the development of more precise treatments and interventions. Precision medicine combines information about individual characteristics, including genetic, health behaviors and environmental exposures to deliver more tailored individual treatments (Ginsburg and Phillips, 2018). Precision public health integrates precision medicine with population-based strategies to increase disease prevention and control (Khoury et al., 2016; Khoury and Galea, 2016). The promise of both fields is to provide the right treatment or intervention to the right individual or population at the right time; however, the promise of greater precision in both fields is yet to be fully realized. In combination, precision medicine, precision public health and DNA-based population screening hold promise to fuel novel, tailored individual and targeted population-level treatments and interventions, while addressing health care disparities (Murray et al., 2018). However, the success of these approaches depends on diverse communities across the United States actively participating (Ginsburg and Phillips, 2018).

The challenge is that many communities have been both historically under-represented in, and abused by, biomedical research in the name of science and medical care (Washington, 2006). Because of this history, many community members distrust biomedical research. Other barriers to engaging diverse community members include lack of awareness of, or comfort with research, and structural barriers such as finances, time, and transportation (Clark et al., 2019). To support DNA-based population screening and advance precision medicine and precision public health, communities historically underrepresented in biomedical research need to be engaged in a manner that fosters trust and inclusivity. To help achieve this goal we present a model of engagement marketing for social good as a framework for supporting community members’ engagement in biomedical research. We acknowledge that attitudes, practices, and approaches on the part of those leading biomedical research needs to be addressed to support engagement. The model we propose provides an initial step for how researchers can frame problems and work with community members using an engagement-focused lens.

One research program that takes a different approach to enrolling participants in a longitudinal cohort is the *All of Us* Research Program (*All of Us* Research Program Investigators et al., 2019). *All of Us* aims to enroll 1,000,000 people that represent the diversity of the United States to drive innovations in biomedical research and precision medicine treatments. Central to the program’s values-driven approach is acknowledging past abuses while working through trusted intermediaries to raise awareness and promote engagement. A key aspect of *All of Us* is engagement with communities that have been underrepresented in biomedical research to help build a relationship with the program that supports informed decisions about enrollment and retention (Richardson-Heron and Cantor, 2019).

Engagement entails active and intentional collaboration with stakeholders (e.g., patients, community members, advocates, health care providers (Chudyk et al., 2018)) to foster connection, interaction, and a long-term bidirectional relationship. The science supporting the benefit of engagement for enrollment and retention in large cohort studies is in its nascency. To help advance the field of engagement, we adapted a conceptual model of engagement marketing from the commercial marketing field and are applying it in our work as an *All of Us* Engagement and Retention Innovator Awardee. We begin by describing commercial engagement marketing, explain how engagement marketing can be translated for social good, describe the conceptual model, and illustrate how we are applying it in our co-creation process to design, develop, deliver, and evaluate digital experiences. These experiences co-created with diverse community members, and other *All of Us* stakeholders, such as health care providers, aim to engage and retain members of communities underrepresented in biomedical research. The engagement marketing for social good model has the potential to inform other efforts focused on DNA-based population screening.

### Engagement marketing from a commercial marketing perspective

Commercial marketing defines consumer engagement as the strategic relationships fostered by an organization or brand, reciprocated by the consumer, and sustained through continuous interactions that supersede the traditional consumer-brand transactional relationship (Harmeling et al., 2016). Engagement marketing is an approach rooted in social exchange theory that leverages the dynamic consumer-organization relationship to advance marketing objectives. Engaged consumers are voluntary co-creators, facilitators, recruiters, and collaborators in developing and executing the organization’s marketing functions (Hollebeek, 2011; Hollebeek, 2016). Engaged consumers are cognitively, emotionally, and behaviorally invested in the success of a brand or organization and their allegiance positions them as valued collaborators rather than mere participants in an economic transaction (Hollebeek, 2011). Engagement marketing values the contributions and active participation of consumers, shifts control from marketers to consumers, and results in consumers becoming engaged and educated intermediaries for a brand or campaign (Harmeling et al., 2016; Burrus et al., 2021).

Evidence indicates that engagement marketing leads to outcomes important to marketers in commercial sectors. Consumers who are engaged with the brands they purchase are more likely to spend more money per transaction, and companies that engage consumers experience an increase in net earnings (Kumar and Pansari, 2016; Alvarez-Milán et al., 2018). Furthermore, commercial engagement strategies can help sustain commerce in downward-spiraling economies (Kumar and Pansari, 2016), increase brand loyalty (Dwivedi, 2015), and endow organizations with valuable feedback on strategy to improve the brand (Dwivedi, 2015; Venkatesan, 2017). These findings suggest that translating and applying engagement marketing to the social good context may also influence behaviors and motivate change for the benefit of individuals and communities in a variety of contexts, such as health and safety, the environment, and social activism (Burrus et al., 2021).

### Engagement marketing from a social good perspective

Previously, we proposed engagement marketing principles can be adapted and applied to health contexts to motivate and empower people to enact prosocial behaviors (Burrus et al., 2021). Engagement marketing, when applied to increasing the social good, involves making a deliberate effort to engage communities with an organization and mission that might not otherwise happen organically (Harmeling et al., 2016). Organizations that typically focus on increasing the social good include non-profits, community organizations, public health departments, and federal, state, and local agencies. Engagement marketing builds relationships, gives a voice to, and fosters collaboration with stakeholders to transform their insights into impactful engagement experiences that motivate and empower them to act to increase the social good. These actions may include making an informed decision, changing a behavior, or joining an effort that promotes social good (Harmeling et al., 2016; Burrus et al., 2021). Engaging communities that have been underrepresented in biomedical research is central to building a diverse research cohort and ensuring that health disparities are not perpetuated by *All of Us*. We view engagement with diverse communities, and the desire to ensure health disparities are not perpetuated as a form of social good, that is, using engagement to enhance health and well-being on a population scale (Mor Barak, 2018).

### Engagement marketing for social good: A conceptual model

Our proposed conceptual model is one that can be applied to many social problems. In the context of *All of Us*, an engagement marketing approach positions participant-volunteers, and other program stakeholders, as active collaborators who will work with us to design, develop, and evaluate digital experiences to support program engagement. This approach may foster trust and transparency that could reduce barriers to participation for members of communities historically underrepresented in biomedical research and the community organizations and health care providers who serve them. Over time, this collaborative approach may be instrumental in supporting ongoing, long-term, impactful engagement across the *All of Us* participant journey.

**FIGURE 1:**
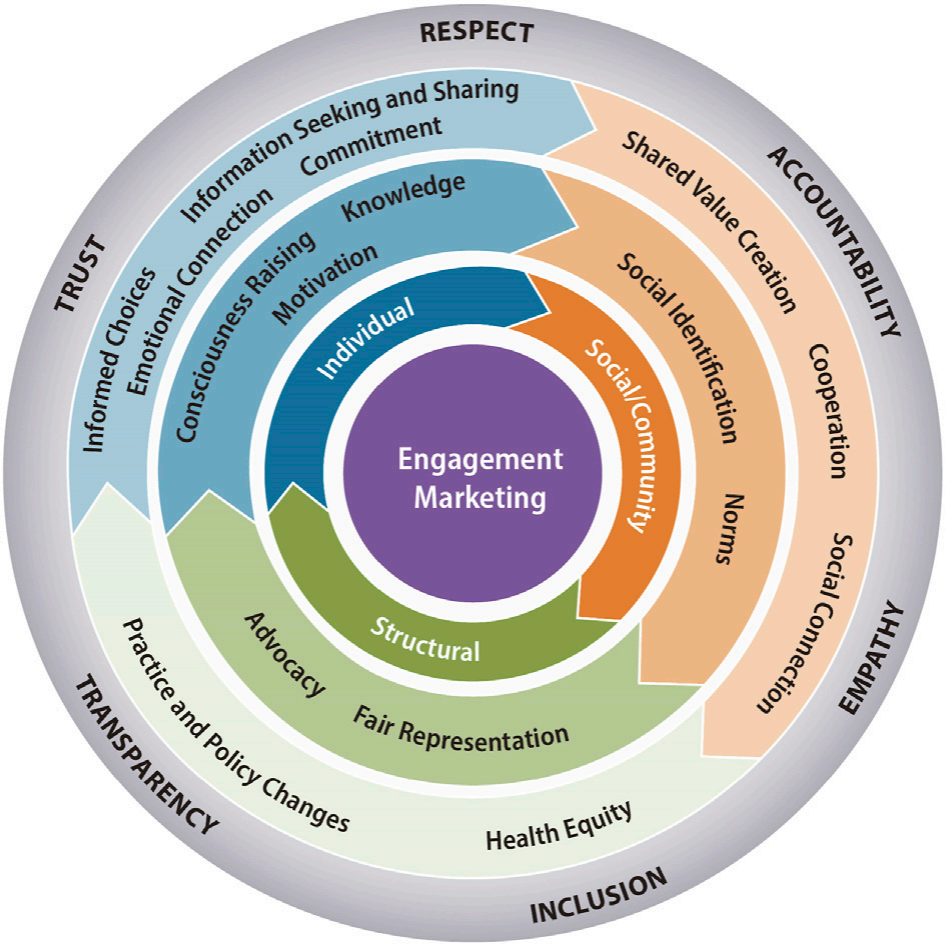
Engagement marketing for social good.

Figure 1 shows our engagement marketing conceptual model to promote the social good. First, we propose that engagement marketing for social good must account for multiple levels of influence, including individual, social/community and structural levels that could impact prosocial behavior and engagement as shown by the different colored sections of Figure 1. Second, we specify potential processes that support change and outcomes at each level, as shown by the colored rings that align with each level. Third, we propose that engagement marketing is driven by values that guide a different type of relationship between community members and researchers, as indicated by the gray ring. Researchers are responsible for upholding these values to foster a different type of relationship.

**Individual-level processes.** Individual-level processes are central to an engagement marketing approach because engagement marketing strategies may fortify a person’s psychological, emotional, and behavioral connection with a social cause (Brodie et al., 2011). Ongoing interactions can increase knowledge and perceived value, and ultimate impact of social good programs. Educated community members become equipped to pass along their knowledge of programs and positive experiences interacting to others, thus expanding the reach and involvement in the program (Hollebeek, 2016). Furthermore, community members’ knowledge and experience aid in the development, management, and dissemination of a cohesive program narrative. Sharing in the distribution of knowledge and information, community members become educated intermediaries who spread program information through their personal and social networks (Harmeling et al., 2016).

Engagement marketing strategies may also prove beneficial for motivating individuals to participate or contribute their own resources to a social cause. Cognitive engagement is a psychological state in which the individual is motivated to advance their relationship with an organization with the expectation that the experience will have greater benefits than costs. (Alvarez-Milán et al., 2018). Positive, memorable, and beneficial experiences may motivate and empower individuals to contribute resources otherwise unattainable to organizers—specifically, network assets, persuasion capital, knowledge, and creative ideas—which may amplify and support program engagement over time.

Engagement with social programs can also raise consciousness about the socio-structural barriers and other sources of oppression contributing to injustices and inequities barring many from better health (Freire, 2000). From an engagement marketing for social good perspective, as individuals become aware of opportunities to reduce inequities through activities perceived as within their realm of control and influence, they may be more likely to participate in developing solutions.

**Individual-level outcomes.** Engagement marketing strategies can cultivate, reinforce, or strengthen a relationship with a social cause. An individual’s perception of having an emotional connection to a program is foundational to their engagement (Kumar and Pansari, 2016). Engagement marketing strategies can help establish and sustain emotional ties to a social cause through reciprocal commitments and ongoing and meaningful social exchanges (Alvarez-Milán et al., 2018).

As community members learn about a cause, become emotionally connected, and motivated they may be more likely to seek out and share information about a social cause. A key outcome predicted in our application of engagement marketing to the social good, information seeking and sharing may be related to involvement in genomics research (Dijkstra et al., 2012). At the individual level, engaging with a social cause may support informed decisions related to taking action to support the effort (Forsythe et al., 2019).

**Social and community level processes.** Social and community level processes are central to an engagement marketing approach, because the approach relies on social exchanges (Hollebeek, 2016). A social exchange approach is consistent with *All of Us* which seeks to develop a longer-term relationship with participants and program stakeholders by exchanging value and promoting collaboration between participants and researchers (Richardson-Heron and Cantor, 2019). Social and community contexts that an individual identifies with can play an important role in shaping openness to engagement with research (American Psychological Association, 2021). Many studies indicate racial or ethnic groups may share levels of awareness, perceptions, or norms surrounding genetic testing and research, perhaps owing to the historic exploitation of African Americans in the Tuskegee syphilis study (e.g., Fairchild & Bayer (1999)) or Native Americans being pressured to demonstrate their heritage through blood and DNA (e.g., Tallbear (2013)). There are also regional differences in awareness and attitudes surrounding genetic testing, sometimes over and above racial or ethnic identification (Jonassaint et al., 2010). Group-level identities and norms provide possible avenues for communicating about the benefits of research involvement which underscores the importance of involving diverse community members in the development of experiences that support engagement with *All of Us*.

**Social and community level outcomes.** From an engagement marketing for social good perspective, potential outcomes related to social and community level processes include creating greater social connection, cooperation, and shared value creation. Because engagement marketing relies on building a relationship with community members and listening to their ideas and concerns, social connections are created by their active participation and co-creation of solutions (Harmeling et al., 2016). This process may also foster shared values and strengthen the capacity of community members to participate in research and achieve collective impact (Porter and Kramer, 2011). Cooperative behavior is a potential outcome of engagement marketing at this level because involving diverse stakeholders in the process of designing solutions that promote engagement is a way of promoting fairer processes and outcomes (Tyler and Blader, 2000).

**Structural level processes.** An engagement marketing perspective is fundamentally a structural change in the way marketing is typically conducted, because it shifts the communication and control between marketers and priority audiences from unidirectional to bidirectional, and a more relationship-based perspective (Harmeling et al., 2016). From an engagement marketing for social good viewpoint, fair representation of diverse voices ensures that the dialogue and decision making is joint, and inclusive of various viewpoints in developing solutions. This is important because fair representation has the potential to make the outcomes of the process more relevant and useful for community members (Israel et al., 1998; Macaulay, 2017), and potentially actionable by the systems that serve them. As community members, and other stakeholders, become more engaged with a social cause, they may be more likely to use their social capital to advocate for the cause (Putland et al., 2013).

**Structural level outcomes. **To date, there has been no empirical examination of how fair representation and advocacy could lead to structural level changes in practice and policy that support an engagement marketing for social good approach. However, there are influential research institutions in place that are investing in building infrastructures that support practice and policy changes that require engagement, representation, and inclusion of diverse patient, community, and system stakeholders. For example, the Patient Centered Outcomes Research Institute (PCORI) has built a research infrastructure in which the voices of patients, community members, and other health care system stakeholders are central to the research process (Frank et al., 2015). The value of this engaged research approach is currently under study through PCORI’s Science of Engagement Initiative.

**Values-based approach.** A values-based approach is important for all levels of engagement marketing for social good. As shown in the outer ring in Figure 1, we believe transparency, inclusion, empathy, accountability, respect, and trust are key values when engaging with stakeholders, especially community members that have been historically marginalized from the research process. By enacting these values as part of engaging stakeholders, our model addresses important ethical issues raised by scholars who study genomic translation. For example, our model addresses responsive justice defined as “starting with the real-world needs of socially situated groups that experience systematic disadvantage” (Burke et al., 2011, p. 12). In addition, the model encompasses the three component parts of responsive justice: fairness (distributive justice), understanding the views of those who have been under-represented and faced discrimination (recognition) and honoring the obligation as researchers with power to identify injustice and make sure fairness and recognition are achieved (responsibility) (Burke et al., 2011).

### An inclusive process to involve stakeholders for developing digital experiences for *All of Us*

The application of engagement marketing for social good is illustrated through our use of human centered design (HCD) to design digital experiences to engage community members and other stakeholders with *All of Us*. HCD can provide an ethical and effective approach to design products and services for underserved populations by understanding their needs, desires, and experiences (IDEO, 2015; Bartlett et al., 2021). HCD aims to understand the core needs of everyone experiencing or impacted by a problem, and to design with those communities to create solutions rooted in people’s actual needs (IDEO, 2015). Similarly, co-creation can be defined as, “collaborative knowledge generation by academics working alongside other stakeholders” (Greenhalgh et al., 2016). Evidence is lacking regarding the application of an amalgam of popular approaches and processes to product design that considers stakeholders, in this case, the end users as a co-creator throughout the product life cycle. Greenhalgh et al.'s review (2016) found unifying principles of successful co-creation include a systems perspective, framing research as a creative endeavor focused on improving human experience, and attention to governance and processes, which is consistent with our engagement marketing model.

**FIGURE 2:**
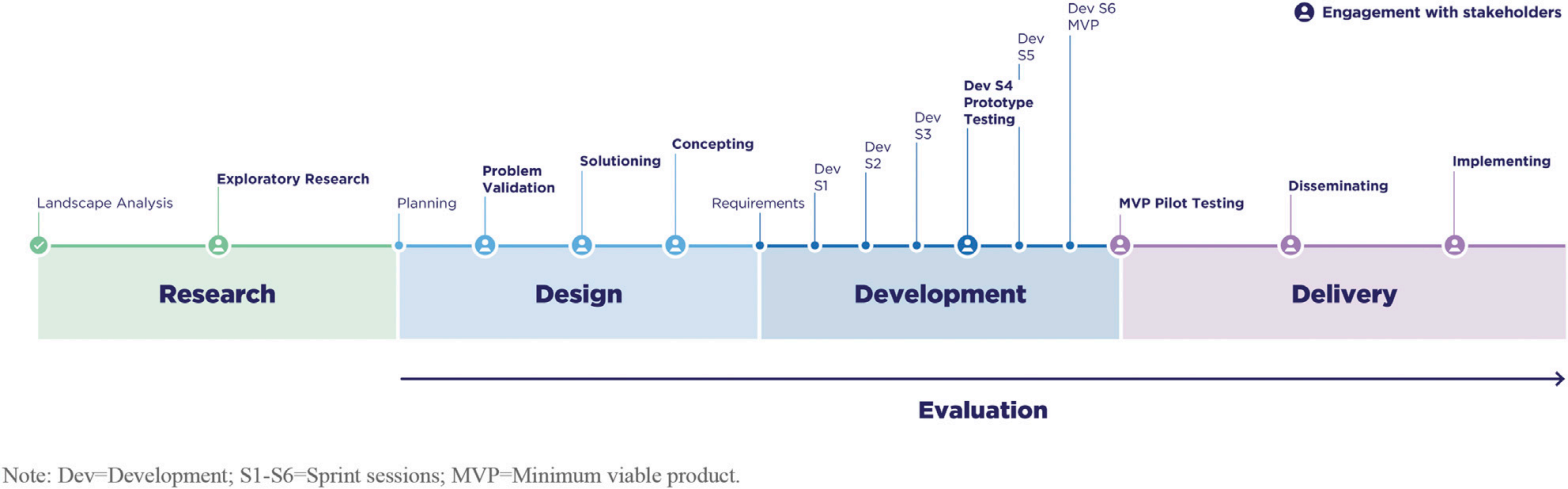
Inclusive approach to engaging stakeholders in the design, development, and delivery of experiences to involve community members and stakeholders with *All of Us*.

To address this gap in applied knowledge, we drew from these unifying principles, industry best practices, and lived experiences to create a systematic process intended to rapidly co-create with a variety of diverse stakeholders to understand and overcome their unique challenges to engaging with *All of Us*. In product design it can be challenging to implement these approaches in a holistic manner; it is not uncommon for stakeholder input to be limited to a single stage in a larger process that limits collaboration and input at critical time points (e.g., concept testing, user testing, and implementation). Our co-creation process is comprised of a design sprint and a development sprint that engages stakeholders at multiple touch points and through a variety of formats (e.g., collaborative workshops, polls, surveys, unmoderated interviews) and techniques that welcome stakeholders as co-creators in designing and developing digital engagement experiences. As shown in Figure 2, we propose to involve stakeholders across the continuum of design, development, and delivery activities that will produce engagement experiences for *All of Us*.

## Conclusion

Beyondmandatednewborn screening, there are no large-scale national programs that implement DNA-based population screening in the United States. *All of Us* is not a screening program but provides one potential model for understanding large-scale collection of genomic information, and how diverse communities across the United States may become involved in DNA-based population screening efforts. As models for DNA-based population screening evolve, greater involvement and trust between historically marginalized community members and researchers will be required. Researchers need to use new models to support greater involvement and engagement in research with communities to make DNA-based population screening successful. We translated an evidence-based engagement marketing approach used in commercial marketing to one that promotes the social good and is being applied to our work as part of the *All of Us* Research Program. This conceptual approach will be continually evaluated using a developmental evaluation approach (Patton, 2010), to understand the extent to which it is successful in promoting engagement, inclusion, collaboration, and trust, which will enable us to refine the process, based on input from the stakeholders who are collaborating with us—furthering value creation. Fostering these outcomes will be essential to advance research and practice for any DNA-based population screening program.
